# Resilience and supporting people living with dementia during the time of COVID-19; A qualitative study

**DOI:** 10.1177/14713012211036601

**Published:** 2021-08-04

**Authors:** Kerry Hanna, Clarissa Giebel, Sarah Butchard, Hilary Tetlow, Kym Ward, Justine Shenton, Jacqueline Cannon, Aravind Komuravelli, Anna Gaughan, Ruth Eley, Carol Rogers, Manoj Rajagopal, Stan Limbert, Steve Callaghan, Rosie Whittington, Lisa Shaw, Warren Donnellan, Mark Gabbay

**Affiliations:** Department of Primary Care & Mental Health, 4591 University of Liverpool, Liverpool, UK; Department of Primary Care & Mental Health, 4591 University of Liverpool, UK; NIHR ARC NWC, Liverpool, UK; Department of Primary Care & Mental Health, 4591 University of Liverpool, UK; NIHR ARC NWC, Liverpool, UK; SURF Liverpool, 4591 University of Liverpool, Liverpool, UK; 130122 The Brain Charity, Liverpool, UK; Sefton Older People’s Forum, Liverpool, UK; Wigan Dementia Action Alliance, UK; Lewy Body Society, Wigan, UK; 8255 North West Boroughs NHS Trust, Warrington, UK; Together In Dementia Everyday (TIDE), Liverpool, UK; Liverpool Dementia Action Alliance, Liverpool, UK; 71283 National Museums Liverpool, Liverpool, UK; Lancashire and South Cumbria NHS Trust, Lancashire, UK; NIHR ARC NWC, Liverpool, UK; EQE Health, Liverpool, UK; Me2U Day Care Centre, Liverpool, UK; Department of Modern Languages and Cultures, 4591 University of Liverpool, Liverpool, UK; Department of Psychology, 4591 University of Liverpool, Liverpool, UK; Department of Primary Care & Mental Health, 4591 University of Liverpool, Liverpool, UK; NIHR ARC NWC, Liverpool, UK

**Keywords:** dementia, older adults, COVID-19, resilience, community care

## Abstract

**Objectives:**

To explore the different factors of resilience for people living with dementia and unpaid carers, in response to sudden changes in care and lifestyle during the COVID-19 pandemic.

**Methods:**

Unpaid carers and people living with dementia were offered telephone interviews in April 2020 to discuss their experiences since the COVID-19 pandemic. Participants were asked about the benefits and challenges of accessing dementia support, as well as coping, symptoms, strategies and impacts. Each transcript was analysed using inductive and deductive thematic analysis by two researchers.

**Findings:**

Semi-structured interviews from 50 participants (*n* = 42 unpaid carers and *n* = 8 people living with dementia) reported protective and risk factors of resilience concerning (1) communication, (2) adaptations, (3) support networks and (4) lifestyle factors and coping mechanisms.

**Conclusions:**

Resilience factors considered both organisational factors for external support, along with individual coping mechanisms. Organisations and social support services should consider resilience factors in future service planning, to better support people living with dementia, or caring someone living with dementia, during times of great stress. The ecological model of resilience established from this research refers to resilience during times of unexpected change in the COVID-19 pandemic; however, it could be considered relevant in other periods of high stress within this cohort.

## Background

Dementia affects an estimated 50 million people worldwide (Alzheimer’s Disease International, 2020) and can negatively impact the person living with dementia and their carers, causing physical and psychological effects (Connell et al., 2001).

Resilience has been described as successful adaptation, competence and functioning in the face of stressful experiences (Bonanno, 2012; Egeland et al., 1993) and has been shown to support people living with dementia and their families in response to caring demands (Gaugler et al., 2007). The terms coping and resilience are, at times, used interchangeably in the literature. However, coping refers to one’s response to aversive situations (Wechsler, 1995), whereas resilience has been used to explain how one copes and ‘bounces back’ following challenging circumstances (Windle et al., 2011). Resilience has multiple concepts that differ depending on the context and cohort described. System/organisational resilience considers the ability of businesses and organisations to respond rapidly to changing environments, whereas social resilience has been described as the ability of individuals and communities to cope with stressful change (Hosseini et al., 2016). An ecological resilience framework considers individual, community and societal resilience to operate across and interact within multiple levels, with further research suggesting a need to move towards more ecological models of resilience in this area of research (Bennett & Windle, 2015; Donnellan et al., 2015; Windle & Bennett, 2012).

An evaluation of the current evidence suggests that resilience is often limited to psychological/trait definitions (Bailey et al., 2013; Cherry et al., 2013; Terracciano et al., 2013). However, researchers have argued that this theory places sole responsibility on individuals to respond resiliently in challenging circumstances and, instead, suggest accountability of resilience should be placed on greater, external factors such as social systems and support (Bonanno, 2012; Donnellan et al., 2015; Southwick et al., 2016). Furthermore, few models exist that look beyond factors associated with the dementia, suggesting a wider exploration of the resilience factors pertinent to this cohort is required.

Since the time of COVID-19, little evidence has robustly explored factors of resilience as reported by people living with dementia or family caregivers. A survey of Italian people living with dementia and family caregivers compared quantitative questionnaire findings from multiple depression and resilience scales, to ascertain a link between high levels of resilience and low levels of depression (Altieri & Santangelo, 2021). However, a limitation of this study is the lack of depth and understanding behind the factors of resilience within this complex subject, from the perspective of the participants. Furthermore, previous models of resilience in dementia have often centred on resilience of the family caring for and coping with the person living with dementia (Deist & Greeff, 2015; Donnellan et al., 2015).

However, life stresses can be multifaceted and one may be required to cope with multiple trepidations simultaneously, adding to the already stressful circumstances of living with dementia, or caring for a person living with dementia. Recent research into the impact of living with dementia during the COVID-19 pandemic has reported a disruption to dementia care and support, increased carer burden and a decline in cognition due to government lockdown measures (Giebel et al., 2020b). Thus, the burden of living with dementia or caring for a person living with dementia, coupled with the recent stresses of living through the global COVID-19 pandemic, presents with a new array of challenges and considerations, for which resilience is critical.

The aim of this study was to explore the different resilience factors reported by people living with dementia and their unpaid carers, in response to sudden changes in care support and lifestyle during the COVID-19 pandemic. Based on current supporting evidence, this research will consider systemic, social and individual contextual factors of resilience, whilst discounting theories of individuals’ personality traits. As part of this research, the development of a new model of resilience was planned, which considers resilience in the face of both dementia and COVID-19 challenges. The rationale for this study is to broaden our understanding of resilience within this cohort and to identify factors that may aid future support planning.

## Methods

### Participants and recruitment

The qualitative study design consisted of in-depth, semi-structured interviews and subsequent inductive thematic analysis. Unpaid carers, ≥18 years old, caring for a person living with dementia at the time of the study or in the past but were still accessing social care or social support services, met the inclusion criteria for this study. People living with dementia with mental capacity to consent were eligible to take part. Recruitment was conducted using convenience sampling nationally, via dementia support organisations, but primarily across the North West Coast of England via social care and social support services. Services advertised the study and contacted eligible participants in the first instance before forwarding details to a researcher. During discussion of the study, prior to recruitment, capacity was assessed by the experienced researcher through the participant’s relayed understanding of the study. Ethical approval was obtained through the University of Liverpool. An approved participant information sheet was emailed to the participants, and re-read to the participants, prior to taking consent. Verbal informed consent was taken before the interview commenced, which was also audio-recorded, as per the approved ethical protocol.

### Data collection

This study is part of a larger programme of research exploring the impact of the COVID-19 pandemic on people living with dementia, and on the family carers (Giebel et al., 2020a). This study describes the responses that specifically related to resilience following the challenges and uncertainty experienced in delivering or receiving dementia care during the pandemic. 50 semi-structured telephone interviews about COVID-19 and dementia care were conducted within the first 2 months of lockdown in the United Kingdom (2020). Interviews were conducted by one author (CG) and a small number were conducted by trainee clinical psychologists, who are experienced qualitative researchers, and who have undergone formal qualitative training previously. The interviewers followed the topic guide to ensure consistency between interviews, and regularly kept in touch throughout the process in case issues occurred or edits were to be made to the topic guide, although, no such issues arose. Interviews lasted up to 50 min, and were audio-recorded. Interviews were conducted by CG and DClinPsych trainees, all of whom were experienced in conducting qualitative research. A topic guide was co-developed amongst the research team, which consists of clinical academics, researchers, service users and carers, to ensure the questions were relevant and central to addressing the research aim. Open-ended questions were carefully framed so as to avoid potential participant bias. The topic guide supported questioning on the participant’s service use before and after the Covid-19 outbreak and governmental restrictions, in respect of coping, symptoms, challenges, benefits, strategies and impacts. The findings in this paper focus on resilience and living with dementia, or caring for a person living with dementia, during the pandemic, which emerged as a prominent topic in response to the line of questioning around new challenges since the pandemic, where participants described methods of adaptation and coping.

### Data analysis

Audio recordings were transcribed into verbatim scripts and each transcript was anonymised and re-read for accuracy. Line-by-line, manual coding was undertaken by the research team (CG, KH, SB, MG, JC, SC, LS) and each transcript was initially coded blindly by two researchers or trainee psychologists. Transcripts were coded immediately, thus subsequent recruitment and interviewing occurred alongside coding, which allowed for a constant comparison of participants and findings. Data were analysed using both inductive and deductive thematic analysis (Braun & Clarke, 2006) by the eight research team members and trainee clinical psychologists. The broad range of professional backgrounds within the research team supported the analysis by ensuring that personal or professional biases did not affect the results. Furthermore, the team met regularly throughout the analysis process to ensure each stage of the process remained focused on the research aim and identified codes were discussed jointly to generate themes35 transcripts were coded using inductive thematic analysis, which were discussed amongst transcribers. This generated the identified themes, with the subsequent transcripts being coded using deductive thematic analysis to complement those themes.

### Public involvement

One person living with dementia and three (former) unpaid carers formed part of the research team, and assisted with conceptualising the study, designing the interview guide, interpreting the findings and with dissemination. This ensured that the study and the interpretation and implications of findings were grounded in the lived experiences of those affected by dementia.

### Findings

#### Demographic characteristics

Table 1 shows the demographic findings. A total of 50 carers (*n* = 42) and people living with dementia (*n* = 8) took part. The sample size aimed to collect a broad perspective around how carers and people with dementia have been affected by the pandemic. It was planned to recruit more carers that people with dementia as cares could also provide insight into the experiences with people in the advanced stages of dementia. Furthermore, the smaller number of people living with dementia recruited to this study is representative of the condition itself, with fewer numbers of suitable research participants compared to the number of recruited family carers. None of the participants were dyads with a pre-existing relationship. The majority of participants were female (76%), and the majority of carers were spouses (55%) living with a person living with dementia (56%), whilst the remaining PLWD resided in their own home alone, with another person, or in a care home. Type of dementia varied amongst Alzheimer’s disease dementia (43%), Lewy body dementia (6%) and vascular dementia (16%), with young-onset dementia without a specific dementia subtype accounting for 12% of participants. Carers lived in a mix of disadvantaged and more affluent neighbourhoods, based on their Index of Multiple Deprivation (IMD) quintile (National Statistics, 2019); however, people living with dementia tended to live in more disadvantaged neighbourhoods.

**Table 1.** Demographic characteristics of people living with dementia and carers.

	Carers (*n* = 42)	People living with dementia (*n* = 8)	Total sample (*n* = 50)
*N* (%)			
Gender			
Female	35 (83.3%)	3 (37.5%)	38 (76%)
Male	7 (16.7%)	5 (62.5%)	12 (24%)
Ethnicity			
White	39 (92.9%)	8 (100%)	47 (94%)
Black, Asian and minority ethnicities	3 (7.1%)	0	3 (6%)
Relation to person living with dementia			
Spouse	23 (54.8%)	—	—
Adult child	19 (45.2%)	—	—
Residence			
Family carer lives with person living with dementia	23 (56.1%)	n/a	23 (46.9%)
The person living with dementia lives at home with another person	0	5 (62.5%)	5 (10.2%)
The person living with dementia lives at home alone	13 (31.7%)	3 (37.5%)	16 (32.7%)
The person living with dementia lives in a care home	5 (12.2%)	0	5 (10.2%)
Dementia subtype			
Alzheimer’s disease	19 (46.3%)	2 (25%)	21 (42.9%)
Mixed dementia	4 (9.8%)	0	4 (8.2%)
Vascular dementia	5 (12.2%)	3 (37.5%)	8 (16.3%)
Lewy body dementia	2 (4.9%)	1 (12.5%)	3 (6.1%)
Young-onset dementia	4 (9.8%)	2 (25%)	6 (12.2%)
Other	7 (17.0%)	0	7 (14.2%)
IMD quintile			
1 (least disadvantaged)	5 (13.2%)	0	5 (11.1%)
2	14 (36.8%)	0	14 (31.1%)
3	8 (21.1%)	1 (14.3%)	9 (20%)
4	4 (10.5%)	3 (42.9%)	7 (15.6%)
5 (most disadvantaged)	7 (18.4%)	3 (42.9%)	10 (22.2%)
Mean (SD), (range)			
Age	60 (+/−8.8) (36–74)	63.6 (+/−6.5) (50–71)	60 (+/−9) (36–74)
Years of education	15.9 (+/−3.8) (11–26)	13.5 (+/−2.6) (11–17)	15.5 (+/− 3.7) (11–26)

#### Thematic analysis

Four themes emerged relating to resilience: (1) communication, (2) adaptation, (3) support networks and (4) lifestyle factors and coping mechanisms. Factors that influenced resilience were described as either pre-dating the COVID-19 pandemic, or occurring in the face of the pandemic. These themes are described below in relation to protective factors of resilience (that supported the person living with dementia or the family carer) and as risk factors (that failed to/ineffectively supported the person living with dementia or the family carer). Resilience was influenced by individuals’ experiences, or through their interaction with social support services. These services include formal, paid home care, out-of-home day care centres and peer support groups.

#### Ecological model of resilience development

**Figure 1.**
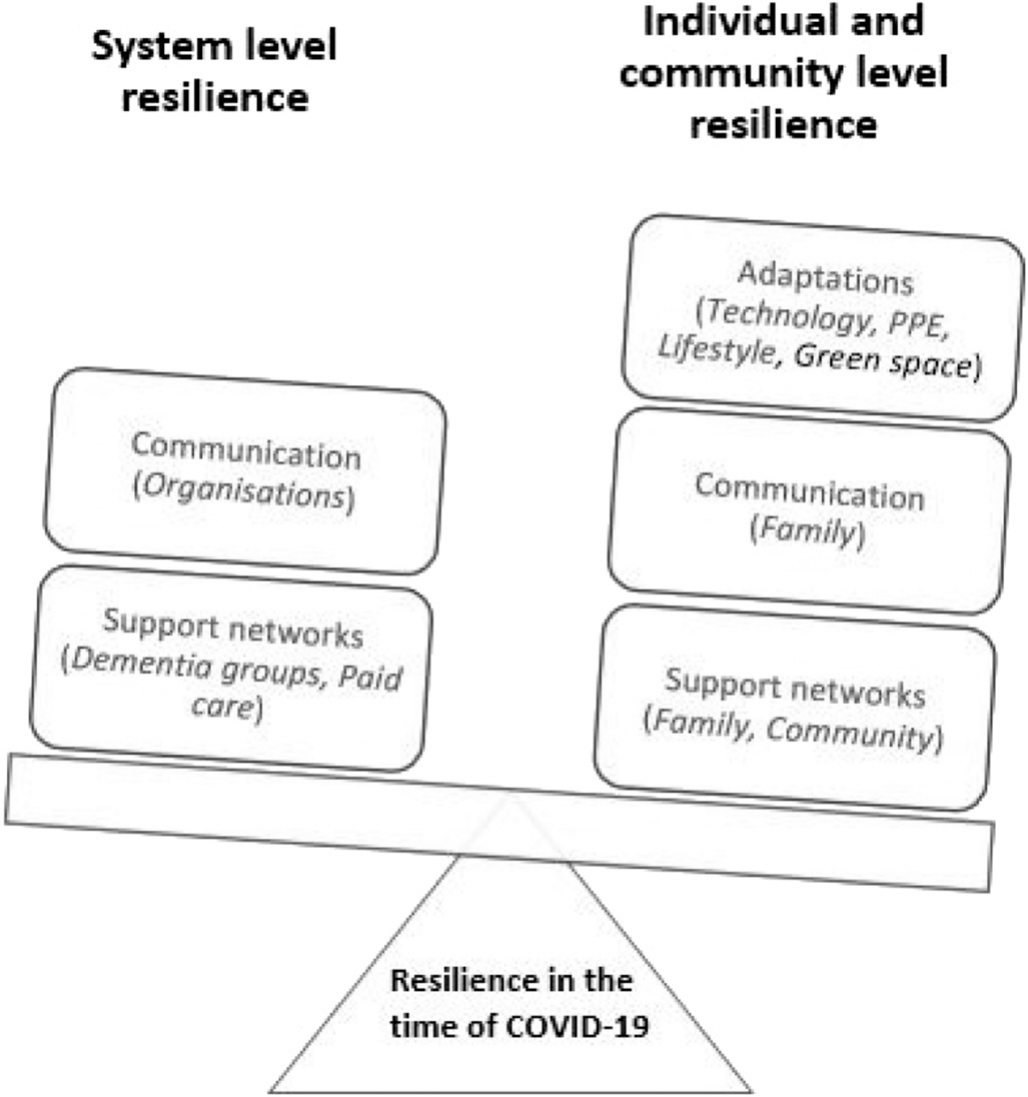
Model of resilience for people living with/caring for a person living with dementia in the time of COVID-19. System resilience consisted of effective and continuous communication among services and people living with dementia/carers. Support networks and paid care that remained active during the time of COVID-19 acted as protective factors of resilience. Individual/community level resilience consisted of effective communication links and active support networks with family and community members. Adaptations to using technology and PPE were mainly reliant on carer support or previous experiences those living with dementia. Good weather and access to green spaces further acted as a protected factor of resilience during lockdown through the opportunities it provided people in accessing social spaces.

Figure 1 shows an ecological model of resilience, for people living with dementia and their family carers in the time of COVID-19, inclusive of the above factors separated by system or individual-level resilience. The model was developed for the purpose of clearly visualising the study findings. One author (WD), a clinical psychologist with expertise in resilience of carers for people living with dementia, provided consult in the model development, which was further refined collectively through research team discussions. Figure 1 shows an imbalance of individual and system level resilience factors, with greater ownership placed on individuals during this time. Within this research, resilience has been conceptualised as an outcome that has been facilitated by resources or factors, and may exist on a scale of varying degrees of resilience as opposed to a binary outcome of present or absent (Johnston et al., 2015).

### Theme 1: Communication

Effective communication ensured that important, practical information was shared by organisations and support/health services during the pandemic, including guidance on social distancing and accessing basic necessities, as well as specific dementia support. Communication links appeared to protect or strengthen resilience as it provided the family carer or the person living with dementia with security in knowing that someone was looking out for the needs. Likewise, the family carers described reassurance in making the correct caring decisions or adaptations for their household based on clear information communicated to them in the early stages of the pandemic, and were further comforted in knowing that they could easily contact organisations if required due to established communication links.

‘(the social worker)’s still in contact with us, she’s the one that suggested the zoom and she’s contacting us on a regular basis, sending us messages asking if we’re ok, do we need any support and also a lot of us have had nurses from the hospital who have followed us the way from diagnosis… they’re still there if we really got stuck’. **Female carer (spouse), Interview 26**

Furthermore, good communication among family caregivers allowed one another to express when the burden was becoming overwhelming, and thus, take a step back, in a continuous cycle of offering and relinquishing care.

‘I think we’re coping as a family unit quite well really, I support my sister she supports me. If there’s something that she’s, if she’s finding it really difficult one day…I’ll say go home, just go, go and do your own thing, I’ll deal with my mum and then she would do the same with me’. **Female carer (spouse), Interview 28**

In contrast, resilience was limited where carers and the person living with dementia experienced poor or absent communication between services, both before and during the time of the pandemic. Descriptions of clinical decisions being made through inadequate communication between the practitioner and the person living with dementia highlighted the importance of effective communication as a protective factor of resilience, adapted to the individual’s needs, and the impact this has on future resilience in terms of trust and reassurance.

‘I took (mum) to this appointment and I had her main carer with me… he (practitioner) didn’t want to speak to either of us at all he only wanted to speak to my Mum and he just said “how did you manage the patches” and she said “oh well yes I think I was alright”. My Mum doesn’t know, it’s a waste of time asking her that sort question when someone’s got dementia… so they put her on a higher dose of (medication) and I think she’d only been taking it 3 days and she got really sick’. **Female carer (daughter), Interview 30**

### Theme 2: adaptations

#### Technology

For many people living with dementia, using technology as a means of communication became an essential form of support during the pandemic. Resilience was described where people living with dementia were able to adapt to using digital communication, and benefitted from sustaining contact with previous activity groups and hobbies. This further enabled socialising and seemed particularly pertinent where fears emerged that those living with dementia were at risk of forgetting their friends and family without regular contact.

‘with things like Zoom and Skype and of course the good old telephone, especially Skype and Zoom because it’s visual, you can interact with the people, you’re seeing them it’s not just audible. I found that extremely helpful’. **Person living with dementia (male), Interview 8**

Factors that influenced the ability to adapt to digital communication included an experience of using this technology before the pandemic, and instances where their pre-existing support groups assisted the person living with dementia in utilising this new method, moving many of the previously enjoyed group activities to an online format.

‘none of them (services) are available as face-to-face so (person living with dementia) can’t go to groups or day-care. However (dementia support group) are being very good and being quite creative in what they’re doing in terms of support without the groups, they have material, do video calls from time to time with some of the people who work there, particularly those who have animals because my wife’s fond of animals’. **Male carer (spouse), Interview 3**

However, some people living with dementia were unable to adapt well to using technology and as such were unable to benefit from remote forms of support and socialising. Key factors that prevented people living with dementia from using technology included no previous internet access installed in the home, which was more common with older people living with dementia, and the requirement of others to be present and support them in using the technology, as they were unable to do this independently due to their dementia.

‘the care home set up Skype but because (person living with dementia) is so poorly now it really doesn’t work apart from one of the nurses taking her own initiative. Last week, because (person living with dementia) was alert, she (nurse) went and grabbed the iPad and suddenly surprisingly she text me and said can I Skype now. So that was quite good but… she stayed with (person living with dementia) and held the iPad because he can’t do it for himself’. **Female carer (spouse), Interview 6**

#### Personal protective equipment (PPE)

Unpaid carers often described the increased efforts they and their family had to undertake to support the person living with dementia during lockdown. Adaptations included using PPE when the carer was entering the home of the person living with dementia, and limiting the time that family could spend with the person living with dementia. Although adaptations provided protection and a sense of control over the situation, thus supporting resilience, it was acknowledged that this form of support resulted in the person living with dementia missing out on personal interaction and emotional support as a result of the purely practical nature of the care. The use of PPE, at times, caused further confusion for the person living with dementia in comprehending this new adaptation to their care.

‘it’s been quite intense now for us…my sisters are both not working at the moment so they’re taking it in turns to go each day with dad) but obviously we’ve got concerns about, we don’t want to transmit anything to him so we’re not staying for very long…they’re wearing gloves and masks and we’re not sitting down and having a cup of tea…we’re bringing a meal…and make sure the washing is done and all of those practical things. But we can’t interact with him in the same way’. **Female carer (daughter), Interview 44**

### Theme 3: pre-existing support networks

#### Family support

Pre-existing support networks, established before the pandemic, played a significant role in supporting resilience towards the stresses and rapid changes in lifestyle that occurred during the pandemic. A supportive family network lessened carer burden through reliance on other family members for practical and emotional support.

‘I think that if I wasn’t in the position I am with having family and people to talk to…it would be very very hard and I guess…it’s not so much the practicalities I think it’s more to do with the emotional impact of it really’. **Female carer (daughter), Interview 44**

Further accounts described a supportive family network that formed during the time of COVID-19. The adverse circumstances of COVID-19 appeared to act as a catalyst for family unity and resilience. Family members made changes to their current lifestyle, often taking on greater caring roles, to support the needs of the person living with dementia during the pandemic.

‘my brother has had to move in from (area name)…’cause he’s been working from home… he’s had to move his home office in with my father just to help with meals and with prevention of contamination as much as we can’. **Female carer (spouse), Interview** 4

Contrasting views of unpaid carers, overburdened with the additional demanding caring responsibilities during the pandemic, without the ability to rely on other family members for support as they too were in lockdown, strengthened this notion that supportive networks protect resilience factors in times of stress. Complex family relationships and external stresses in addition to those brought on by caring for people living with dementia during the pandemic meant that carers did not always feel comfortable relying on family members for support, hindering resilience and feelings of coping.

‘(person living with dementia) is very lucky that he has that input from them (daughters) but because my children aren’t (person living with dementia)’s…I don’t feel as though I can ask them too much. And one of my daughters has had a lot of depression and so I’m sort of walking on egg shells a little bit…it’s not just (person living with dementia) it’s everybody else’s challenges in this time’. **Female carer (spouse), Interview 33**

#### Dementia support and activity groups

A notable protective factor of resilience in the time of COVID-19 was the ability to maintain active participation in pre-existing dementia support groups, and partaking in group activities, albeit adapted to lockdown and social distancing restrictions. Support groups were described as day centre activities or external dementia support group meetings. Participants highlighted that the purpose of such groups was less concerned with the activity, but with socialising and sharing experiences that supported one another in times of stress. Thus, it was an important factor for many to maintain contact, where possible, with their support groups during the times of high stress brought on by COVID-19, in order to sustain a resilient attitude to the stressful circumstances around them by sharing practical advice and emotional support.

‘we’ve got a WhatsApp group that we send messages through and if somebody’s having a particularly bad day we send through just messages of hope really. One or two of us have got partners in residential care so that’s quite good, we’re able to say if any of the homes have been diagnosed with having the virus and how that’s going. So (support group) was extremely supportive in terms of being able to talk to people in the same position’. **Female carer (spouse), Interview 26**

Where support groups were no longer able to meet, or the person living with dementia was unable to partake in digital forms of socialising, it was noted that regular telephone calls from supportive organisations or healthcare providers, in the few instances where this was offered, provided reassurance and comfort to those isolating at home.

‘you can ring people for a chat or if you need help with delivery or your medication bringing, there are numbers available for that, and people will do that…at the minute we are managing but I know there is that service available’. **Female carer (daughter), Interview 1**

Moreover, a risk factor of resilience was described as a lack of access to all available dementia support before COVID-19. This was exemplified where the dementia diagnosis occurred just before lockdown, meaning that the person living with dementia was unable to form those supportive links that could benefit them and reduce carer burden in lockdown.

‘my dad was diagnosed relatively recently…we were offered the opportunity to go on a post diagnosis course… that was incredibly helpful to me because it helped me to understand a lot of things …it was good for him because he got to meet other people who are in similar situations… we were offered the opportunity to go onto other courses… now we haven’t started either of those (because of lockdown)’. **Female carer (daughter), Interview 44**

#### Community support

Likewise, people living with dementia and their carers identified supportive networks within the community, described as friends and neighbours who adopted an active role in looking out for one another. Furthermore, many of the dementia support groups formed close friendships outside of the group meetings, which played a vital role in offering a deeper level of emotional support during stressful periods. As with family support, this provided protection and security as the community ensured each other had the basic necessities, such as food and medicines that they needed, thus supporting resilience in the time of the pandemic.

‘I’ve got really good neighbours so if they don’t see me for a few days they’ll ring from across the road literally…and my next-door neighbour’s the same and if I’m out shopping, if I get out I’ll say…do you want anything…. So we sort of look out for each other so there’s quite a good community support round here’. **Female carer (spouse), Interview 26**

Conflicting views described a lack of resilience in the absence of supportive community networks. It was observed that a lack of contact from friendship groups during this stressful period negatively impacted the people living with dementia and their carers.

‘I missed the groups and the support that we get and we do phone each other we want to hear a voice we get fed up of texting and “WhatsApping” so… we had a video link the other day just to see each other’s faces really. So it’s that personal contact, even though you’re on the phone it’s seeing them and being near them…that’s gone out the window’. **Female carer (spouse), Interview 26**

#### Paid home care

The addition of paid home care during the pandemic formed a protective factor of resilience in cases where the care was supportive and reliable. Accessing paid care allowed the person living with dementia to live independently whilst further reducing overburdened feelings of stress for the unpaid carer, thus allowing them to maintain a positive and proactive mental attitude.

‘well I think I’m extremely fortunate to have such good carers, I mean I suppose it defines my life in a way but I’m able to live an independent life because I can rely on them, I know that they will come, I know they won’t let me down… I have been able to lead a life and go out and I haven’t had to worry’. **Female carer (daughter), Interview 27**

### Theme 4: lifestyle factors and coping mechanisms

#### Lifestyle factors

Evidence of previous coping mechanisms to support the person living with dementia and their carer in times of stress emerged from the transcripts signifying protective factors of resilience. Exercise, in the form of walking and running, was one example of a coping mechanism, which previously supported resilience in times of stress and was adopted during the time of COVID-19 to sustain a positive mindset.

‘this lockdown sort of occurred with the fairly warm weather…so we’ve been going out we’re running at the moment every other day which is I think good for our mental health and its giving him a challenge… the brain tells you to stop and you just have to say shut up and push through that and he finds that very very difficult but I discovered that if I set off on the faster part of the run with him behind me he tries stopping but he won’t let me get very far in front of him… So it’s about tactics to get him through’. Female carer (spouse) Interview 31

Further evidence from people living with dementia who previously lived contentedly with minimal socialisation showed signs of resilience during the time of COVID-19. As the lockdown restrictions had a minimal impact on their usual routines, they did not report the same high levels of stress as seen in other accounts of highly active and sociable people living with dementia. Thus, one’s lifestyle and level of social engagement prior to the pandemic appeared to coincide with their resilience and ability to cope with the new lockdown and social distancing restrictions.

‘I’m quite happy on my own at home, I’ve got my friend…I’ve got my little dog and I’ve got my iPad and I just keep trying to research on my own and keep my mind active, I’m not really a person that needs to be with a group and to be chatting and talking and laughing, I’m quite more just want to read and learn stuff’. **Person living with dementia (female) Interview 20**

#### Access to green space

A common theme that emerged from the transcripts was the topic of good weather and access to nature and green spaces. This supported resilient accounts where socialisation and exercise were made possible, whilst it was suggested that one must possess an internal ‘capability’ to cope, which was reinforced by external factors and the positive effect these had on one’s mental well-being.

‘I’ve got a garden so that’s fine, I’m fine actually, I feel guilty that I can’t do more really because I’m over 70 and that limits you (due to government shielding advice) but physically and mentally I’m perfectly capable… I realise at the moment the best thing one can do is just keep out of danger’. **Female carer (daughter), Interview 27**

In contrasting accounts, people living with dementia who resided in flats or apartments, or in homes with no access to a garden, appeared to be more impacted by lockdown restrictions as they no longer had the freedom to leave the house.

‘the apartment that I live in, there is a very large car parking bay it’s about 70 feet long and 30 feet wide, steel railings around it, huge gates… so I feel like I’m in a prison yard, so I just go round and round that looking through the bars, I feel like I’m in prison’. **Person living with dementia (male) Interview 8**

Thus, limited access to outdoor, green spaces during the pandemic could be a risk factor for reduced resilience and coping.

## Discussion

Recent evidence has portrayed adverse circumstances for people living with dementia during the pandemic, including loss of care support, new or increased reliance on others, increased loneliness and an increased rate of contracting the COVID-19 virus due to inability to follow social distancing guidance (Brown et al., 2020; Giebel et al., 2020b). However, this is the first study to our knowledge that has explored resilience in people living with dementia, or caring for a person living with dementia during the COVID-19 pandemic.

Findings from this study described communication, adaptations, support networks and lifestyle and coping mechanisms as factors of resilience during the pandemic. Coping mechanisms, a previous experience of using technology and paid care remained consistent for some during the pandemic and supported resilience. In contrast, increased family or community support and a loss of social support groups, along with the requirement for some participants to adapt to using PPE and technology during the pandemic, described the necessary efforts made to support resilience. These findings support earlier reports that resilience in dementia caregiving is dynamic (Gaugler et al., 2007) and highlight to clinicians and policy makers the factors deemed helpful during this time of great stress and uncertainty.

The current study findings suggest that resilience in the time of COVID-19, for people living with dementia and their carers, hinged on organisational and societal factors, whereby effective support networks reinforced resilience, and a lack of organisational support, ineffective or lacking communication and a loss of social support services hindered resilience. This notion supports previous findings that psychological or traits-based resilience models place too much responsibility for change on individuals, whilst promoting the notion that ecological models provide a holistic approach to resilience, placing such responsibility on services and policy (Donnellan et al., 2015). On a psychological level, individuals discussed adaptations and coping mechanisms such as technology and exercise that supported notions of resilience, which concurs with previous evidence where these factors are described as useful coping mechanisms that also support cognition in dementia (Brown et al., 2015; Rabinowitz et al., 2010). Access to outdoor green spaces played an importance role in protecting resilience during the pandemic, supporting subsequent findings from a multi-national survey amongst the general European population, which showed a relationship between mental health and blue/green spaces during the pandemic (Pouso et al., 2021). However, the size and quality of indoor pace was not discussed in our study, albeit access to the internet. Amerio et al. (2020) reported found a link between indoor space and mental well-being during the pandemic; however, this research included participants who began working from home due to the lockdown measures, which differs greatly from the current study population, offering a possible explanation for this finding.

However, successful coping strategies were not recommended by organisations, but instead were specific to the individuals’ abilities and experiences before COVID-19, or learned from peer support during the pandemic where remote communication was possible. This finding may explain the variation in resilience among participants and highlights a general lack of support for this cohort during the pandemic, resulting in higher levels of stress amongst the family carers and the people with dementia. With greater weight placed on individual-level resilience, as opposed to system-based resilience, we propose the ecological model of resilience shown in Figure 1, adapted from the study findings.

Evidence in non-dementia-specific research suggests resilience is a multi-dimensional concept, consisting of environmental, social, economic and community components, and as such, focus is shifted from the individuals, and towards individuals’ engagement with systems (Popay et al., 2018). Therefore, a key recommendation from this current research is for organisations and social support systems to consider resilience factors in future care/support plans, in order to better protect people living with dementia and their family carers in the event of stressful experiences. Furthermore, amongst the closure of day centres and the inability for support groups to meet face-to-face, paid home care played a role in supporting resilience in unpaid carers, by reducing stress and burden, along with the benefits this brought to the people living with dementia, as this was one of the only social support services that somewhat remained throughout the pandemic. The few remaining forms of social support significantly aided people living with dementia, and the family carers, in maintaining greater levels of resilience, showcasing the importance of social support services in improving quality of life for people living with dementia and their carers.

Moreover, recommendations have been suggested to better support people living with dementia and carers during the pandemic, including a more person-centred approach to dementia care, and the use of technology to access dementia care remotely, avoiding physical contact and virus transmission (Cheung & Peri, 2020). The current study found that technology played an important role in supporting people living with dementia and the family carers during the pandemic but was less frequently described as an independent task carried out by the people living with dementia, and instead required the support of the carer. This finding has been specifically identified in the face of the pandemic, whereby people living with dementia have been forced to adapt quickly to technology when they were perhaps not technologically confident beforehand (Hill et al., 2015). Furthermore, the care adaptations described inhibited the previous level of emotional support gained from face-to-face contact, in place of the much-needed practical support during the pandemic. As new evidence is emerging promoting the use of technology in dementia care following the COVID-19 pandemic (Cuffaro et al., 2020), this study shows that such adaptations should be adopted with caution, whilst always considering the abilities of individuals, using person-centred care models (O’Shea, 2020).

Effective communication and contact with organisations and support networks during the pandemic supported resilience in the time of COVID-19, as this allowed for feelings of support and trust in services and organisations, of which participants increasingly relied upon during this time of stress. Communication between family members caring for people living with dementia has been included in previous dementia-specific models as a factor of resilience (Deist & Greeff, 2015; Donnellan et al., 2015, 2017). However, our finding that communication with organisations and support services appears pertinent to the time of the pandemic.

Our findings report two levels of resilience at play: (1) living with dementia, or caring for someone living with dementia, and (2) the additional stress of living through the global pandemic. Therefore, although these findings relate to COVID-19 research specifically, it is possible that these factors may apply to other major life stresses experienced by this cohort. The ecological model of resilience (Figure 1) highlights the individual factors of communication, adaptations and support networks that can help to foster resilience amongst family carers and people living with dementia; however, greater system level support and communication is further required to lighten the individual burden of fostering resilience during the time of this pandemic, or in future, stressful events.

## Limitations

Due to the convenience sampling method of this research, although purposive, few people of Black and Asian minority ethnicities and rare dementias were included in this study and so their views on resilience have arguably not been captured fully. Future research in the field of dementia and resilience should aim to target and recruit those from minority backgrounds to ensure inclusive views are collected. A smaller number of people living with dementia participated compared to family carers, however, as this is a group often excluded from research due to mental capacity; a small sample will still allow for views to be represented that may otherwise be missed. The study may be limited as interviews could not be conducted face-to-face, although the research team were well experienced in qualitative interviewing and did not observe any issues during the interview process.

## Conclusions

A lack of resilience was described where system resilience was lacking and the people living with dementia, and their family carers, were unsupported by services and organisations. Therefore, organisations and social support services should consider resilience factors in future service planning and aim to better support people living with dementia and family carers during times of great stress. The ecological model of resilience established from this research refers to resilience in living with dementia, or caring for someone living with dementia, during times of unexpected change and high stress in the COVID-19 pandemic, which is going to last into the foreseeable future. Moreover, this can also be relevant to other periods of high stress in dementia.
